# Embedding Resilience Activities Across an Undergraduate Pharmacy Degree: An Educational Improvement Initiative and Cross-Sectional Evaluation

**DOI:** 10.3390/pharmacy14050111

**Published:** 2026-08-03

**Authors:** Abdullah Al-Juhaishi, Nilushi Karunaratne, Annie Chen-Chen, Anisha Kaur Sandhu, Kirsten Galbraith, Betty Exintaris

**Affiliations:** 1 Faculty of Pharmacy and Pharmaceutical Sciences, Monash University, Melbourne, VIC 3052, Australia; abdullah.jaafar@monash.edu (A.A.-J.); nilushi.karunaratne@monash.edu (N.K.); annie.chen-chen@monash.edu (A.C.-C.); kirstie.galbraith@monash.edu (K.G.); 2 School of Pharmacy, Monash University, Bandar Sunway 47500, Malaysia; anisha.sandhu.1@unimelb.edu.au

**Keywords:** resilience, undergraduate curriculum, interfaculty collaboration, pharmacy

## Abstract

Resilience, the capacity to recover from adversity, manage stress, and maintain well-being, is vital for pharmacy students to manage academic stress, clinical challenges, and future professional demands. Despite its recognised importance, pharmacy curricula often lack structured resilience education. This pilot study aimed to design, implement, and evaluate resilience-focused activities within Monash University’s Bachelor of Pharmacy (Hons)/Master of Pharmacy program. This study employed a descriptive cross-sectional educational evaluation design using a single post-intervention survey to explore student perceptions following participation in curriculum-integrated resilience activities within the Skills Coaching program. Sessions used reflective frameworks and interactive small-group activities facilitated by staff and pharmacists across the Australian and Malaysian campuses. In 2022, 1061 students participated, with feedback response rates between 26 and 40%. Students reported perceived improvements in their understanding of resilience following participation in the sessions. Likert-scale responses indicated improved understanding of resilience and highlighted strong engagement with case discussions and reflective exercises. Findings from this pilot inform curriculum refinement, assessment design, and faculty development to support sustainable integration of resilience education in pharmacy programs. This work also supports the lead author’s doctoral research to develop a comprehensive resilience curriculum for undergraduate pharmacy education.

## 1. Background

Universities are increasingly responsible for nurturing not only academic excellence but also the emotional and psychological well-being of their students [1]. In health professional programs such as pharmacy, this responsibility is amplified by rigorous training demands and the high-stakes nature of future professional practice. Resilience, commonly defined as the capacity to adapt positively to adversity, manage stress, and maintain a sense of control, has emerged as a critical non-cognitive skill for both academic success and long-term professional sustainability [2,3]. Beyond supporting academic performance, resilience may help protect students and practitioners against burnout, psychological distress, and professional attrition, all of which are increasingly recognised concerns within healthcare education and practice.

The concept of resilience in education highlights strengths such as optimism, perseverance, and self-efficacy as protective factors in challenging contexts [4,5]. In parallel, educational psychology models emphasise reflective learning, emotional regulation, and developmental progression, suggesting that resilience is not static but can be taught, practised, and strengthened over time [6,7]. Professional identity formation theories similarly emphasise resilience as an important component of becoming a healthcare professional, enabling students to navigate uncertainty, mistakes, feedback, and emotionally demanding situations encountered during training and practice [2].

Studies have consistently associated higher resilience with improved well-being, smoother university transition, and lower psychological distress among students [8]. Resilience benefits personal welfare and significantly impacts students’ ability to engage in complex tasks, confront challenges, and maximise opportunities [9]. Recognising this, university curricula addressing student health and well-being should operate at the system, program, and subject levels [8,10], acknowledging that resilience is shaped not only by individual capacity but also by supportive learning and work environments [11,12,13]. Consequently, universities are increasingly encouraged to address student well-being at system, program, and subject levels rather than relying solely on individual coping strategies [8,10].

Although resilience training has gained traction in medicine and nursing, pharmacy education continues to lag behind [3]. Few pharmacy curricula feature structured and long-term resilience programs for students [14,15]. Most existing efforts are limited to single-session wellness initiatives or extracurricular workshops, which often lack theoretical coherence, evaluative rigour, and alignment with program learning outcomes. This gap is also reflected in programs, where existing well-being initiatives provide a foundation, but opportunities remain for a more structured and longitudinal approach [3,16,17].

Globally, pharmacy education has evolved significantly in response to technological advancements, workforce demands, and students’ mental health needs. Contemporary pharmacy programs increasingly emphasise active and experiential learning approaches [18]. Recent studies highlight stress, academic overload, and inadequate well-being support as major concerns for pharmacy students. There is a positive correlation between academic resilience, student well-being and retention [19,20]. These challenges, which were further highlighted during the COVID-19 pandemic, reinforce the growing interest in resilience-related support within higher education [21,22], further reinforcing the necessity of resilience training in higher education.

Despite recognising resilience as a core competency, few pharmacy programs have structured resilience training in their curricula [23]. Literature reviews and surveys highlight the lack of integrated frameworks scaffolded across academic years and tailored to student development [24]. The Global Resilience Team (GRiT) has promoted resilience within pharmacy professionals by fostering a community focused on well-being and professional development [25]. These initiatives are particularly important in addressing the systemic pressures that contribute to burnout, a major challenge that can diminish resilience and adversely impact both personal well-being and professional practice. Implementing such initiatives presents challenges, especially for academics focused on specialised fields with limited experience in holistic approaches [26]. To incorporate resilience-building, education must expand beyond professional knowledge to address personal development [27]. McDonald et al. (2013) advocated for emphasising the ‘self’ domain, which is critical for fostering resilience against professional and personal challenges [28].

To implement resilience-building education, academics must adapt their teaching methods and organisational structures to include well-being initiatives, such as promoting student resilience at the program level. Success depends on academic staff’s participation in modifying their design, teaching, and assessment methods [29,30]. Resilience features in global frameworks, including the World Health Organisation (WHO) European policy for health and well-being, which identifies it as crucial for health and societal capacity, and the United Nations Sustainable Development Goals (SDGs 4 and 5), which emphasise inclusive education and well-being for human resilience [31,32]. Resilience is essential for healthcare professionals, but pharmacy education rarely structures training accordingly [33]. Prior to this pilot initiative, the Monash University Pharmacy Program did not have a unified resilience curriculum implemented consistently across its Australian and Malaysian campuses. This study piloted a coordinated resilience curriculum across both campuses through the Skills Coaching program. Current efforts are fragmented and lacking in systematic evaluation. This study is set within the Skills Coaching program across the Bachelor of Pharmacy (Hons)/Master of Pharmacy degree at the Monash Australian campus, with the long-term aim of embedding the curricula across both the Australian and Malaysian campuses.

This study aims to:

1. Develop resilience activities specifically tailored for pharmacy students at various academic levels.
2. Assess the feasibility of delivering this curriculum through the existing Skills Coaching program at Monash University’s Australian (MUA) and Malaysian (MUM) campuses.
3. Explore students’ perceptions regarding the usefulness, relevance, delivery, and perceived educational value of the resilience activities.

## 2. Methods

### 2.1. Setting

The Faculty of Pharmacy and Pharmaceutical Sciences at Monash University offers a comprehensive five-year Bachelor of Pharmacy (Hons)/Master of Pharmacy program. Initiated in 2017, this dual-degree program is available at Monash University’s campuses in Australia and Malaysia [29]. A distinctive feature of this program is its unique instructional approach, in which basic science is taught within the contextual framework of either a patient or a medicine, thereby fostering interdisciplinary integration. Beyond acquiring content knowledge, the curriculum emphasises skill development through deliberate connections to real-world applications. Established educational principles include active learning methodologies, a concentrated emphasis on employability, and the incorporation of experiential learning. A prominent strategy employed is Skills Coaching, defined as a structured and supportive process wherein an ‘expert’ guides a learner toward achieving a professional goal, specifically the acquisition or enhancement of a relevant skill set [29].

The Skills Coaching program is integrated with the pharmacy undergraduate curriculum, extending over the initial four years of the course. This coaching methodology is systematically designed to promote student growth, development, awareness and accountability [29]. The program includes student reflections, individualised feedback, and focused skill-based team meetings, each lasting 60 min and occurring six times annually. As students advance through the course, the coaching system evolves to accommodate their changing needs and emerging professional identities. A notable aspect of the coaching program is the strategic assignment of coaches to align with students’ developing professional identities. In the first year, academic staff are coaches. From the second year onwards, pharmacy students transition to working with registered pharmacists as their coaches. This phase aims to broaden students’ perspectives on their future professions by exposing them to the essential skill set required in the field. Coaching in the third and fourth years seeks to further enhance professional awareness and facilitate a deeper analysis of the knowledge, experience, and feedback gained during experiential learning placements. This progressive coaching model ensures that students are thoroughly prepared for their evolving roles as pharmacists-in-training.

With a focus on skill development and small group size, the Skills Coaching program was an ideal place to embed the resilience curriculum within the degree. Integrating the topic of resilience into the coaching program over four years also provided students with the opportunity to track their personal development and build a resilience e-portfolio through submitted reflections.

### 2.2. Curriculum Description

The project team consisted of academics with varied backgrounds and at different stages of their careers. The group included two early-career researchers and two senior academics, one of whom served as a Director and Global Lead for Advanced and Speciality Practice at the International Pharmaceutical Federation (FIP) Hub and played a pivotal role in formulating global pharmacy workforce objectives. Two members of the resilience team were part of the global group of resilience experts (GRiT). Among the team, two were pharmacists, and all members were involved in teaching within the pharmacy program. One team member was based at Monash University Malaysia, and three academics were affiliated with Monash University Australia.

In 2021, an internal faculty education grant was received to support the development of the resilience curriculum. The funds were used to hire a practitioner educator (hospital pharmacist) to support curriculum design and development.

The resilience curriculum was developed by an expert team that drafted key features outlining the structure and content of the curriculum. This draft included a year-wise breakdown of activities and goals aimed at skill development related to resilience-related skill development. Each year (Y1 to Y4) featured specific sessions or workshops focused on introducing resilience concepts, reflecting on personal challenges, and identifying resilience aspects to improve over the 12-month period. This approach ensured progressive skill-building and self-assessment of resilience throughout the educational and early professional journeys (see Appendix A). As this was a single-group educational intervention embedded within the curriculum, no assignment or allocation to different study conditions was undertaken. Although reported with consideration of TREND recommendations, several elements (e.g., lack of comparator group and baseline data) were not applicable due to the study design [34]. Challenges included scheduling the sessions around an already packed curriculum, standardising delivery across campuses, and ensuring that facilitators felt confident with sensitive discussions. These were addressed through collaboration with coaching leads, incorporating feedback loops, and developing facilitator guidance materials.

### 2.3. Educational Activity

A resilience curriculum was embedded in the Skills Coaching program of Monash University’s Bachelor/Master of Pharmacy program. These small group sessions, supported by reflective learning, provide a framework for resilience-building activities. The curriculum addressed gaps in pharmacy education by implementing scaffolded training based on positive psychology and professional identity theory, with activities tailored to students’ developmental stages.

- Year 1: Introduction to resilience and self-reflection.
- Year 2: Cognitive reframing and self-talk strategies.
- Year 3: Flexible thinking and uncertainty management.
- Year 4: Learning from mistakes and recovering from errors.

The sessions were tailored to pharmacy experiences using examples such as medication errors and exam stress. Each activity included discussion guides, reflection templates, and real-world scenarios to foster application beyond the classroom (See Appendix B, Appendix C, Appendix D and Appendix E). Feasibility was measured through educational objectives, including increased understanding of resilience and improved confidence in applying the strategies used. Outcomes were assessed using Likert-scale surveys and qualitative feedback, capturing cognitive and affective changes. The skills coaches supported consistency and provided insights into student engagement and the perceptions of the sessions.

#### 2.3.1. Evidence-Based Design of the Activity

Curriculum development was based on theoretical constructs from positive psychology and educational psychology. The core principles of positive psychology, including optimism, self-efficacy, and a growth mindset, guided session themes such as self-talk, flexible thinking, and learning from mistakes [15,35]. These findings align with key protective factors in resilience literature, such as adaptive coping and reflective [36]. Reflective practice used Borton’s developmental framework (“What? So what? Now what?”), which builds metacognition and emotional awareness for professional resilience [37]. These theories provide a rationale for embedding resilience as a dynamic developmental capability rather than a static trait. This pilot study lays the groundwork for developing a robust, evidence-based resilience curriculum within pharmacy programs. The development of resilience activities is centred on evidence-based design features, including collaborative learning and reflective practice, as well as evidence-based strategies for building resilience, such as self-assessment surveys. After the initial meeting with the practitioner educator, two members of the expert team met with the practitioner educator four times for consultation and feedback before presenting a draft of the resilience curriculum to the wider group. Upon further feedback and refinement of the curriculum, the expert group met with the Skills Coaching leads for years 1–4 to describe the curriculum and discuss logistics for the pilot study. In years 1–3, the resilience pilot was embedded into Skills Coaching sessions in the 1st semester, while in the 4th year, the resilience curriculum was embedded into the second semester. In semester 1, the 4th-year students are on placement and return to the campus for what is known as ‘Back to Base Day’, where they participate in a 2 h resilience workshop.

The resilience curriculum was integrated into the Skills Coaching program to introduce resilience to 1st-year students and teach them strategies for developing resilience as they progress to practising pharmacists. Students reflected on resilience sessions using Borton’s ‘What, So What, Now What’ framework [37]. Students received personalised feedback from their Skills Coaches using the ‘Keep, Start, Stop’ framework [38], and each resilience session included guided reflective prompts (Appendix B, Appendix C, Appendix D and Appendix E). The pilot curriculum was implemented across both campuses during 2022. As a pilot curriculum-wide implementation delivered to all enrolled students, comparator groups and random allocation were not feasible during the initial implementation phase.

#### 2.3.2. Framework

The resilience curriculum was informed by complementary theoretical perspectives, including positive psychology, educational psychology, and professional identity formation [14,39]. Together, these frameworks informed the design and delivery of the resilience activities across the pharmacy program.

1. Positive Psychology

Positive psychology emphasises the cultivation of strengths and adaptive traits such as optimism, gratitude, self-efficacy, and perseverance as protective factors in the face of adversity [40]. These constructs informed the curriculum’s emphasis on self-talk, goal-setting, and cognitive reframing, particularly in the Year 2 and Year 3 activities. Resilience was positioned not as the absence of distress, but as the capacity to grow and maintain purpose in the midst of challenges.

2. Educational Psychology

Educational psychology contributed reflective and developmental models that shaped how resilience was taught and measured. Borton’s reflective model (“What? So what? Now what?”) was used to scaffold students’ self-assessment, emotional processing, and critical thinking. Concepts such as scaffolding, metacognition, and zone of proximal development underpinned the longitudinal design of the curriculum, allowing for progressive complexity from foundational awareness (Year 1) to professional application (Year 4).

3. Professional Identity Formation

Resilience is conceptualised as an essential element in the development of professional identity within the healthcare sector, where students are required to adeptly manage errors, uncertainty, and emotional complexities. The Year 4 activity, which concentrated on the recovery from professional errors, explicitly incorporated insights from literature on emotional resilience and moral distress in pharmacy and medical practice. This approach was instrumental in equipping students not only to endure but also to engage ethically and reflectively in challenging real-world situations.

By anchoring the curriculum to these theoretical foundations, each session moved beyond surface-level well-being to engage in deeper cognitive, emotional, and professional competencies. These frameworks also informed the evaluation tools, with survey items designed to assess perceived self-awareness, adaptability, and confidence, which are the hallmarks of resilient learners and practitioners.

### 2.4. Evaluation of the Pilot

This study employed a descriptive cross-sectional educational evaluation design using a single post-intervention survey to explore student perceptions following participation in curriculum-integrated resilience activities within the Skills Coaching program. As an initial curriculum implementation project embedded within routine educational delivery, the evaluation was designed as a pragmatic first-stage descriptive assessment focusing on feasibility, implementation, and student perceptions rather than intervention effectiveness. Although the resilience activities were scaffolded across the curriculum, the evaluation did not follow a longitudinal cohort of students over time. Instead, cross-sectional feedback was collected separately from students within each academic year during the 2022 implementation period. A cross-sectional survey was administered to all students during the final session to assess their perceptions following participation. A uniform agreement-based Likert response format was used across survey items to simplify administration within the curriculum evaluation setting. The primary outcomes of this study were student perceptions of usefulness, understanding, and session quality. All enrolled students in Years 1–4 were eligible. Participation in the intervention was mandatory as part of the curriculum, while survey completion was voluntary. Participants were not randomly assigned; all students received the intervention as part of the curriculum.

The anonymous survey consisted of 10 Likert-scale questions and one question relating to content and structure. Five questions were related to the perceived usefulness of the activities in each session, two questions related to pre- and post-understanding of the topic of resilience, and three questions related to the organisation and overall quality of the session. The question related to how the resilience session could be improved. The same survey was used for all year levels.

### 2.5. Statistical Analysis

Given the descriptive evaluative purpose of this pilot educational initiative, analyses focused primarily on frequencies and percentages describing student participation (see Table 1) and their perceptions across year levels rather than hypothesis testing or inferential comparisons. Likert-scale responses were summarised using frequencies and percentages for each survey item and stratified by year level (P1–P4), as presented in Table 2, Table 3 and Table 4. No inferential statistical tests or subgroup comparisons were conducted as part of this analysis. To protect participant confidentiality and encourage honest responses, data were collected anonymously. Blinding was not applicable, as all participants received the intervention, outcomes were self-reported, and the unit of analysis was the individual student. Survey analyses were conducted using available responses for each survey item. Respondents were not excluded from the dataset based on incomplete survey responses, and missing data were not imputed.

## 3. Results

### 3.1. Student Perceptions of the Resilience Activities

As shown in Table 1, the resilience curriculum was administered to 1061 undergraduate pharmacy students across all academic years in 2022. Sample size was determined by the total number of enrolled students across the Australian and Malaysian campuses. Although responses were obtained across all academic year levels and both campuses, demographic and campus-specific characteristics were not collected. Therefore, the representativeness of respondents relative to the broader student population could not be formally assessed. Feedback was collected from 322 students, yielding an overall response rate of 30.4%. The response rates varied among the cohorts, with the highest participation recorded among Year 2 students (40.1%), followed by Year 1 students (32.0%). In contrast, lower but comparable response rates were observed among Year 3 (25.7%) and Year 4 (25.6%) students. These data provide an overview of the distribution of student engagement with curriculum evaluation across different year levels. Baseline measures were not collected as part of the study design.

#### 3.1.1. Students’ Views of the Perceived Usefulness of the Resilience Activities

Table 2 summarises students’ perceptions of the usefulness of resilience activities across year levels. Overall, the responses indicated high perceived usefulness and relevance, with the majority of students reporting agreement or strong agreement that the activities were beneficial, relevant to their future practice, and supportive of their understanding and development of resilience. Positive perceptions were consistently observed across cohorts for all survey items, including content relevance, reflective learning, understanding resilience, and the value of integrating multiple activity components (e.g., introduction, activities, and discussion). This suggests that the curriculum was well-received at all stages of the program.

**Table 2:** Students’ views on the perceived usefulness of resilience activities.

	P1 (*n* = 94)					P2 (*n* = 89)					P3 (*n* = 75)					P4 (*n* = 64)				
Survey Item	Strongly Agree (%)	Agree (%)	Neither Agree Nor Disagree (%)	Disagree (%)	Strongly Disagree (%)	Strongly Agree (%)	Agree (%)	Neither Agree Nor Disagree (%)	Disagree (%)	Strongly Disagree (%)	Strongly Agree (%)	Agree (%)	Neither Agree Nor Disagree (%)	Disagree (%)	Strongly Disagree (%)	Strongly Agree (%)	Agree (%)	Neither Agree Nor Disagree (%)	Disagree (%)	Strongly Disagree (%)
Please indicate the perceived usefulness of the resilience activity	46 (49)	41 (43)	7 (7)	0	0	45 (47)	30 (31)	8 (8)	4 (4)	2 (2)	34 (36)	33 (35)	7 (7)	1 (1)	0	20 (21)	35 (37)	8 (8.5)	1 (1)	0
The content of the resilience activity is relevant to my future work	71 (76)	23 (24)	0	0	0	61 (69)	21 (22)	4 (4)	2 (2)	1 (1)	50 (67)	23 (24)	2 (2)	0	0	43 (67)	19 (20)	2 (2)	0	0
The activities helped me to reflect deeper on my ability to develop resilience	51 (54)	38 (40)	3 (3)	2 (2)	0	48 (51)	23 (24)	12 (12)	3 (3)	3 (3)	35 (37)	31 (32)	7 (7)	2 (2)	0	17 (18)	32 (34)	14 (15)	1 (1)	0
The activities helped me to gain an understanding of resilience	59 (62)	29 (30)	5 (5)	0	1 (1)	49 (52)	27 (8)	10 (1)	1 (1)	2 (2)	35 (37)	29 (30)	9 (9)	2 (2)	0	17 (18)	30 (31)	14 (15)	3 (3)	0
The various aspects of the activity (introduction, activities, discussion) worked well together to help me learn about resilience	50 (58)	36 (38)	8 (8)	0	0	43 (45)	31 (32)	11 (11)	3 (3)	1 (1)	28 (29)	37 (39)	8 (8)	2 (2)	0	21 (22)	32 (34)	10 (10)	1 (1)	0

#### 3.1.2. Pre- and Post-Understanding of Resilience and Session Quality

As demonstrated in Table 3 and Table 4, students across all academic levels exhibited a marked transition from a limited understanding of resilience before the session to enhanced comprehension following their engagement in the skills-based activities. Students reported perceived differences between their retrospective pre-session and post-session understanding of resilience following participation in the activities. Simultaneously, students provided positive evaluations of the session in terms of its organisation, overall quality, and adequacy of time allocation. Collectively, these findings suggest a beneficial educational impact of the resilience session on students’ perceived understanding, corroborated by favourable assessments of the session design and delivery.

### 3.2. Organisation and Overall Quality of the Session

Overall, students across all year levels reported generally positive perceptions regarding the relevance, usefulness, and organisation of the resilience activities. Most respondents agreed that the sessions supported their understanding of resilience and encouraged reflective consideration of professional challenges.

Similarly, students reported favourable views regarding the organisation of the session. In P1, 71% (67) strongly agreed, and 28% (26) agreed that the session was well organised. In P2, 68% (64) strongly agreed, and 18% (17) agreed. In P3, 46% (43) strongly agreed and 24% (23) agreed, whereas in P4, 43% (40) strongly agreed and 20% (19) agreed. Neutral responses remained relatively low across cohorts, ranging from 1% (1) in P1 to 8% (8) in P3, with disagreement reported by 0–1 student in each cohort.

When asked to rate the overall quality of the activity, most students provided positive responses. In P1, 68% (64) strongly agreed and 30% (28) agreed that the activity was of high quality. In P2, 55% (52) strongly agreed, and 28% (26) agreed. In P3, 38% (36) strongly agreed, and 31% (29) agreed, whereas in P4, 28% (26) strongly agreed and 34% (32) agreed. Neutral responses ranged from 2% (2) in P1 to 7% (7) in P3, whereas disagreement remained minimal across cohorts (0–3%, corresponding to 0–2 students).

Overall, while most students across all cohorts evaluated the session positively, a gradual reduction in the proportion of strongly positive responses was observed across cohorts.

**Table 3:** Student views on pre- and post-understanding of the topic of resilience.

	P1 (*n* = 94)					P2 (*n* = 89)					P3 (*n* = 75)					P4 (*n* = 64)				
Survey Item	Strongly Agree (%)	Agree (%)	Neither Agree Nor Disagree (%)	Disagree (%)	Strongly Disagree (%)	Strongly Agree (%)	Agree (%)	Neither Agree Nor Disagree (%)	Disagree (%)	Strongly Disagree (%)	Strongly Agree (%)	Agree (%)	Neither Agree Nor Disagree (%)	Disagree (%)	Strongly Disagree (%)	Strongly Agree (%)	Agree (%)	Neither Agree Nor Disagree (%)	Disagree (%)	Strongly Disagree (%)
Prior to this skills session, my understanding of the topic of resilience was limited	14 (15)	20 (21)	35 (37)	23 (24)	2 (2)	16 (17)	21 (22)	18 (19)	24 (25)	10 (10)	10 (10)	14 (15)	21 (22)	27 (29)	3 (3)	10 (11)	15 (16)	15 (16)	17 (18)	7 (7)
At the end of this skills session, my understanding of the topic of resilience remained limited	10 (10)	10 (10)	7 (7)	27 (29)	40 (43)	17 (18)	7 (7)	8 (9)	21 (22)	36 (38)	11 (12)	6 (6)	8 (9)	19 (20)	31 (33)	10 (11)	9 (10)	4 (4)	18 (19)	23 (24)

**Table 4:** Student views on organisation and overall quality of the session.

	P1 (*n* = 94)					P2 (*n* = 89)					P3 (*n* = 75)					P4 (*n* = 64)				
Survey Item	Strongly Agree (%)	Agree (%)	Neither Agree Nor Disagree (%)	Disagree (%)	Strongly Disagree (%)	Strongly Agree (%)	Agree (%)	Neither Agree Nor Disagree (%)	Disagree (%)	Strongly Disagree (%)	Strongly Agree (%)	Agree (%)	Neither Agree Nor Disagree (%)	Disagree (%)	Strongly Disagree (%)	Strongly Agree (%)	Agree (%)	Neither Agree Nor Disagree (%)	Disagree (%)	Strongly Disagree (%)
The time given to participate in the resilience activity was sufficient	61 (65)	27 (29)	5 (5)	1 (1)	0	52 (55)	29 (31)	4 (4)	2 (2)	2 (2)	39 (41)	29 (31)	7 (7)	0	0	37 (39)	21 (22)	5 (5)	1 (1)	0
Overall, the skills session was well organised	67 (71)	26 (28)	1 (1)	0	0	64 (68)	17 (18)	6 (6)	1 (1)	1 (1)	43 (46)	23 (24)	8 (8)	1 (1)	0	40 (43)	19 (20)	5 (5)	0	0
Overall, please rate the quality of this activity	64 (68)	28 (30)	2 (2)	0	0	52 (55)	26 (28)	7 (7)	2 (2)	2 (2)	36 (38)	29 (31)	7 (7)	2 (2)	1 (1)	26 (28)	32 (34)	6 (6)	0	0

## 4. Discussion

The findings of this study should be interpreted within the context of a descriptive educational evaluation. As no comparator group, randomisation, or prospective baseline assessment was included, the study was not designed to determine causal effectiveness. Instead, the findings provide preliminary insights into the feasibility, implementation, and student perceptions of embedding resilience activities within a longitudinal pharmacy curriculum.

Specifically, this study examined the implementation and feasibility of a pilot structured resilience curriculum integrated throughout the Bachelor of Pharmacy (Hons)/Master of Pharmacy degree at Monash University. This study explores how embedding resilience training within the Skills Coaching program, a longitudinal small-group professional development stream, supports students in developing personal resilience and preparedness for professional challenges [29]. As a pilot educational initiative, the study explored student perceptions of curriculum-integrated resilience activities. The findings from this pilot educational initiative may help inform future longitudinal and controlled studies examining resilience education within pharmacy curricula.

The resilience curriculum will aid in their personal development and help them recognise their individual strengths, weaknesses, coping strategies, and support mechanisms [41,42]. This is also in line with the vision set by the International Pharmaceutical Federation (FIP), which highlights the importance of meeting current needs in pharmacy education by developing a future pharmacy workforce that engages in lifelong learning by identifying their learning needs through self-assessment to recognise their own limitations and act on them as part of personal or professional competencies [43,44].

### 4.1. Comparative Context of the Resilience Curriculum

Resilience training in pharmacy education is limited and often short-term. In health professions education, most resilience interventions are either single-session workshops or optional wellness resources, often lacking integration into the curriculum or developmental scaffolding [11,12,42,45]. For example, some pharmacy schools in the UK and North America have introduced short-term well-being modules or mindfulness training, but these are typically non-assessed, one-off offerings [20,46,47].

The positive student perceptions observed in this study may partly reflect the integrated and longitudinal design of the resilience curriculum. Unlike many previously reported resilience interventions, which are often optional, short-term, or delivered as standalone workshops, the Monash resilience activities were embedded within a compulsory professional development stream (Skills Coaching) across multiple year levels and international campuses [36,42]. This structure enabled iterative learning, progressive skill development, and ongoing reflection supported by pharmacist mentors. Furthermore, the incorporation of authentic pharmacy-related challenges, including medication error scenarios in senior years, may have strengthened the perceived relevance of the activities by connecting resilience development to real-world professional practice. These findings suggest that scaffolded and professionally contextualised resilience curricula may be more meaningful and sustainable than isolated well-being interventions, with potential implications for pharmacy educators and curriculum designers seeking to address student well-being and burnout within healthcare education.

### 4.2. Alignment with Educational Standards

At the national level, the Australian Pharmacy Council’s (APC) Accreditation Standards for Pharmacy Programs (2020) require that graduates demonstrate capabilities related to professional identity, reflective practice, and resilience [48]. Embedding resilience into the Skills Coaching stream directly supports graduate attributes such as personal leadership, adaptability, and emotional regulation. Thus, the resilience program not only responds to local educational policy but also contributes to the global discourse on embedding wellness and self-efficacy in pharmacy education.

Future studies could strengthen the evaluation by incorporating a comparator group, such as students from a pharmacy program without an embedded resilience curriculum, or a cohort that has not yet received the intervention. This would allow comparisons of resilience, well-being, preparedness, and burnout-related outcomes. The use of validated pre- and post-intervention measures, longer-term follow-up, and qualitative interviews would also support a more robust assessment of curriculum impact.

### 4.3. Longitudinal Follow-Up

Importantly, there is scope for the resilience curriculum, which is designed to equip students with essential skills such as stress management, adaptive coping strategies, and maintaining mental well-being, to be utilised by students enrolled in the faculty’s pharmaceutical sciences programs (Bachelor of Pharmaceutical Sciences and Master of Pharmaceutical Sciences) in the future, in addition to embedding it in the pharmacy curriculum. Integrating this curriculum aims to strengthen students’ professional readiness and personal resilience, thereby aligning with the overarching goals of these programs to cultivate competent, adaptable, and well-prepared pharmaceutical professionals capable of navigating the demands of their field effectively [10,49].

Given the developmental nature of resilience, future research should incorporate longitudinal and quasi-experimental designs to track student resilience development across the four-year program. This includes the use of validated resilience measures (e.g., CD-RISC-10, Brief Resilience Scale) at multiple time points: entry into the program, after each yearly intervention, and after course completion. This data will allow the team to assess trends in resilience growth over time and identify the curricular components that yield the greatest sustained impact. Future evaluations may also benefit from including comparator cohorts from pharmacy or health professional programs without embedded resilience curricula to allow for comparative analysis of student perceptions, resilience-related outcomes, and well-being measures across differing educational contexts.

Additionally, post-graduation follow-up surveys will be developed to explore how former students perceive the value of the resilience curriculum in their transition to professional practices. This longitudinal perspective will help determine whether resilience-building activities during university translate into lasting professional competencies and improved workplace well-being.

### 4.4. Educational Implications

The outcomes of this study highlight that embedding resilience into a longitudinal professional development framework is both feasible and well-received, but maximising its impact requires intentional alignment with the wider curriculum. This includes linking resilience concepts to clinical content, experiential placements, and interprofessional activities, as well as incorporating resilience-based competencies into formal assessments of students’ performance. For other pharmacy schools and health professional programs, adopting a similar scaffolded approach while adapting to local contexts, resources, and learner needs could address the well-documented gaps in student well-being support. At the policy level, these findings reinforce the need for accreditation standards and curriculum frameworks to explicitly include resilience and well-being as core graduate attributes, ensuring that the next generation of pharmacists is equipped to thrive in increasingly complex and high-pressure healthcare environments.

### 4.5. Limitations

This descriptive cross-sectional evaluation has methodological limitations restricting causal interpretation. First, standard demographic characteristics were not collected, limiting the ability to assess representativeness and explore potential demographic influences on perceptions. Second, retrospective self-assessment items collected in a single post-intervention survey may have introduced recall and response-shift bias. Consequently, perceived changes in understanding should not be interpreted as objective evidence of intervention effectiveness. Third, the cross-sectional nature prevents assessment of sustained changes in resilience. Additionally, the 30.4% response rate introduces potential non-response bias, as respondents may differ from non-respondents. Finally, as the study was conducted within one institution, findings may be context-specific and not generalisable to other programs, and the absence of validated resilience outcome measures further limits conclusions regarding resilience-related competencies.

A further limitation relates to the evaluation survey design. Although a uniform agreement-based Likert format simplified administration, some item stems were not optimally aligned with this scale. In particular, items asking students to indicate the perceived usefulness or quality of the activity may have been more appropriately assessed using rating-based options. This mismatch may have affected how students interpreted and responded to these items and should be considered when interpreting findings. Future iterations will use response formats better aligned with the construct being assessed, such as quality or usefulness rating scales for evaluative items and agreement scales for statements of agreement.

## 5. Conclusions

This manuscript describes the implementation and descriptive evaluation of a pilot resilience curriculum integrated within the undergraduate pharmacy program across the Australian and Malaysian campuses of Monash University. Overall, students reported positive perceptions regarding the relevance, organisation, and perceived educational value of the resilience activities. The findings suggest that embedding resilience-related reflective activities within an existing professional development framework is feasible and acceptable within pharmacy education.

While the evaluation design limits conclusions regarding effectiveness or long-term impact, the curriculum provides a foundation for future longitudinal and comparative research exploring resilience development in pharmacy students. The findings may also inform other pharmacy and health professional programs seeking to integrate resilience-related learning activities within existing curricula.

## Figures and Tables

**Table 1:** Student participation in resilience curriculum feedback by year level (2022).

Year Level	Total Enrolled Students	Students Providing Feedback	Response Rate (%)
Year 1	297	94	32
Year 2	222	89	40
Year 3	292	75	26
Year 4	250	64	26
Total	1061	322	30

**Figure A1:**
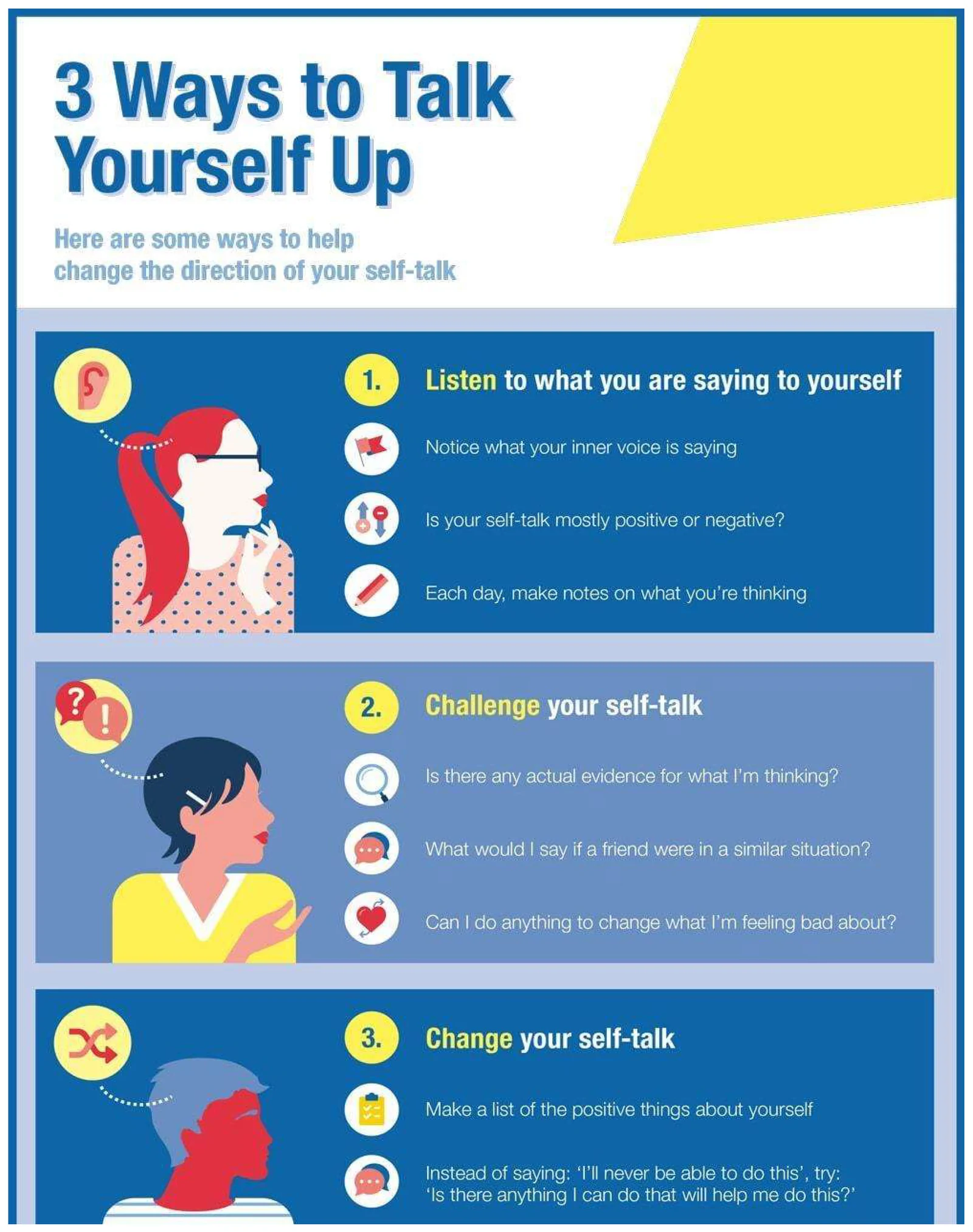
Facilitator Note: Supporting Positive Self-Talk.

## Data Availability

The original contributions presented in this study are included in the article. Further inquiries can be directed to the corresponding author.

## Appendix A

Proposed Resilience Curriculum

Year 1 Orientation

Skill development activities within a designated workshop

- Introduce the concept of resilience
- Define resilience
- Evaluate baseline resilience

Year 1 Skills Session: Reflect on a situation where you encountered challenges as a secondary school student. Objective: Over the next 12 months, identify two aspects of resilience to focus on improving. Skill development activities: To be designed and developed

Year 2 Skills Session: Reflect on a situation where you encountered challenges as a university student. Objective: Over the next 12 months, identify two aspects of resilience to focus on improving. Skill development activities: To be designed and developed

Year 3 Skills Session: Reflect on a situation where you encountered challenges as a student or professional (university/work/placement). Objective: Over the next 12 months, identify two aspects of resilience to focus on improving. Skill development activities: To be designed and developed

Year 4 Back to Base Day Workshop Skill development activities within a designated workshop

- Explore previous resilience
- Explore existing resilience

Year 4 Skills Session: Reflect on a situation where you encountered challenges as a professional (work).

Linked to the Back to Base workshop

Objective: Over the next 12 months, identify two aspects of resilience to focus on improving.

Skill development activities: To be designed and developed.

## Appendix B

First-year skills coach: What is resilience?

Intro: 5 min: Ask each student to brainstorm their definition or synonym for resilience.

Definition: “An individual’s capacity to manage the everyday stress of work and remain healthy, rebound and learn from unexpected setbacks, and prepare for future challenges proactively.” [50]

Choose one of the three videos (depending on what you think will suit your group of students):

1. https://www.youtube.com/watch?v=ASDBJXDNqvc (1:30 min) (accessed on 16 December 2025)
2. https://www.youtube.com/watch?v=G33wqX4A-38 (2:12 min) (accessed on 16 December 2025)
3. https://www.youtube.com/watch?v=yyX6UULJEic (3:02 min) (accessed on 16 December 2025)

Watch the video together, and have a discussion (5–10 min each) on:

- How did your original definition/synonym for resilience differ from what was mentioned in the video:

Box A1 Understanding Resilience. For facilitators: Please ensure students understand that resilience doesn’t just encompass the synonyms but is the ability to recover and bounce back from situations.

- Ability to cope with things when they go wrong, overcoming challenges.
- Being able to withstand or recover quickly from difficult conditions
- Ability to “bounce back” following adversity.

Synonyms: elasticity, strength, perseverance, flexibility, elasticity, adaptable

b. Why resilience is an important skill as a pharmacy student/pharmacist and/or Why having resilience will help in the COVID-19 pandemic as a student and/or pharmacist.

Box A2 Why Resilience Matters. For facilitators: “Resilience positively relates to mental health outcomes, job and career satisfaction, job engagement, enhanced coping abilities and post-traumatic growth; while negatively relating to stress, cynicism, burnout, and emotional exhaustion. Having resilience mitigates burnout and other effects of work-related stress.” [50] For pharmacists: Ability to cope with stress and to empathise with patients, therefore, providing better health and customer care. Adapting and developing new ideas/solutions to deal with potential surges in demand for medications, PPE and ventilators. For students: Ability to adapt to different work/study challenges, thereby preparing for real-life situations. Having resilience allows students to learn from challenges and minimise the effects of negative, stressful situations.

c. What strategies can help to build resilience/what are the characteristics of people with resilience?

Box A3 Practical Ways to Build resilience. For facilitators (Can discuss a few examples here)

- Spend time and energy focusing on situations that you have control over (e.g., working to keep customers safe within the pharmacy) rather than worrying about things you can’t control
- Choose your response—sometimes you can’t control the events that happen to you, but you can always choose how you respond. Choose to remain calm and find a logical solution
- Practise thought awareness—notice how you talk to yourself when things go wrong. If you respond with negative self-talk, try challenging these thoughts
- Set personal goals—when you achieve a goal, even a very small one, this builds your self-confidence
- Strengthen relationships with your friends/colleagues so you can support each other through challenges as a team.
- Develop problem-solving skills—Research suggests that people who are able to come up with solutions to a problem are better able to cope with problems than those who cannot. Whenever you encounter a new challenge, make a quick list of some of the potential ways you could solve the problem.
- Embracing change—Resilient people often utilise these events as an opportunity to branch out in new directions. While some people may be crushed by abrupt changes, highly resilient individuals are able to adapt and thrive.
- Positive thinking/staying optimistic does not mean ignoring the problem in order to focus on positive outcomes. It means understanding that setbacks are temporary and that you have the skills and abilities to combat the challenges you face. Keep in mind that every situation is different. There may be times when positive thinking/staying optimistic is challenging for reasons beyond your control. It is important to recognise that when these situations arise, you seek support from those around you and professional services.

For their next reflection: Reflect on what are/were the challenges transitioning from year 12 to university. From what they’ve learnt on resilience in this session, how can they build resilience through these challenges?

More resources on resilience:

https://www.verywellmind.com/ways-to-become-more-resilient-2795063 (accessed on 16 December 2025)

https://schools.au.reachout.com/resilience?gclid=EAIaIQobChMIlq6h5b6a9QIVBCQrCh2XiAfEEAMYASAAEgKp7PD_BwE (accessed on 16 December 2025)

https://www.youtube.com/watch?v=eHyv_LFXkVU (accessed on 22 November 2025)

https://www.youtube.com/watch?v=G33wqX4A-38 (accessed on 22 November 2025)

https://www.youtube.com/watch?v=bcgyemmOmCA (accessed on 22 November 2025)

## Appendix C

P2

Self-talk and positive thinking

Last year, students were introduced to the concept of resilience.

5–10 min: Recap—ask students to define what resilience is

Box A4 Facilitator Note: Defining Resilience. Definition: “An individual’s capacity to manage the everyday stress of work and remain healthy, rebound and learn from unexpected setbacks, and prepare for future challenges proactively.” [50] Please ensure students understand that resilience doesn’t just encompass the synonyms but is the ability to recover and bounce back from situations.

- Ability to cope with things when they go wrong, overcoming challenges.
- Being able to withstand or recover quickly from difficult conditions
- Ability to “bounce back” following adversity.

Synonyms: elasticity, strength, perseverance, flexibility, elasticity, adaptable

For Facilitators:

This year, we’ll explore self-talk and positive thinking, which are strategies to build resilience and are characteristics of people with resilience. Positive self-talk is the key to developing optimism. Optimism involves learning to think positively about the future, even when things go wrong. It’s about looking objectively at a situation and making a conscious decision to focus on the good. It is important to build resilience and positive thinking, as people are happier, more engaged, succeed more often and are better problem solvers.

5–10 min: Ask students to volunteer and read together the following article (https://schools.au.reachout.com/resilience/optimism) (accessed on 9 June 2025) on self-talk and discuss the following questions:

“Even though you might not know it, you’re already practising self-talk.”

Self-talk is basically your inner voice, the voice in your mind that says the things you don’t necessarily say out loud. We often don’t even realise that this running commentary is going on in the background, but our self-talk can have a big influence on how we feel about who we are.

The difference between positive and negative self-talk

Positive self-talk makes you feel good about yourself and the things that are going on in your life. It’s like having an optimistic voice in your head that always looks on the bright side.

Examples: ‘I am doing the best I can’, ‘I can totally make it through this exam’, ‘I don’t feel great right now, but things could be worse’

Negative self-talk makes you feel pretty crappy about yourself and the things that are going on. It can put a downer on anything, even something good.

Examples: ‘I should be doing better’, ‘Everyone thinks I’m an idiot’, ‘Everything’s crap’, ‘Nothing’s ever going to get better.’

Negative self-talk tends to make people pretty miserable and can even impact their recovery from mental health difficulties. But it’s not possible, or helpful, to be positive all the time, either. So, how can you make your self-talk work for you?

Why should I practise?

The more you work on improving your self-talk, the easier you’ll find it. It’s kind of like practising an instrument or going to sports training: it won’t be easy to start with, but you’ll get better with time.

It might not seem like much, but self-talk is a huge part of our self-esteem and confidence. By working on replacing negative self-talk with more positive self-talk, you’re more likely to feel in control of stuff that’s going on in your life and to achieve your goals.

5–10 min: Give students 5 min to think through these questions and ask a few to share (adapted from https://schools.au.reachout.com/resilience/optimism) (accessed on 16 December 2025):

- Describe a challenging situation you faced where self-talk was involved.
- Was your self-talk positive or negative? Give an example.
- Did you do anything to try to change your thinking? What worked?
- What would you say if a friend were in this situation?

For your next reflection: Reflect upon a time when you hadn’t performed as well as you hoped academically in University.

- Was your self-talk positive or negative? Give an example.
- From what you’ve learnt about optimism/positive thinking today, how could you challenge/change your self-talk?
- What goal could you set for yourself to improve next time?

## Appendix D

P3

Flexible thinking

Last year, we looked at positive self-talk and optimism, characteristics of resilient health care workers.

Another characteristic of resilient people is the ability to think flexibly or the ability to adapt. This year, we’ll look at flexible thinking or adaptability. Thinking flexibly allows you to confidently tackle the unknown or the unexpected and is a key ingredient to developing resilience.

What is flexible thinking?

Box A5 Facilitator Note: Flexible Thinking and Plan B. For facilitators: Flexible thinking allows for multiple solutions to a problem. As things don’t always go to plan, being able to develop alternative plans (Plan Bs) is a vital aspect of resilience. Resilient people understand that there are occasions when we may have to abandon our initial plans and switch to a backup plan (Plan B).

10 min: Ask students to think about and share the following questions (adapted from https://schools.au.reachout.com/resilience/flexible-thinking) (accessed on 16 December 2025):

- What decisions have you had to make today?
- What impacted these decisions and your ultimate choices?
- Did you have to change a plan (e.g., what to have for breakfast, or how you were going to get to school)?

10 min: What are some good strategies for good decision-making? (adapted from https://schools.au.reachout.com/resilience/flexible-thinking) (accessed on 16 December 2025):

Box A6 Facilitator Note: Decision-Making and Resilience. Tips for making decisions

- Don’t let stress get the better of you. Stress can make you feel anxious and cloud your thinking. You might tend to rush your decisions without thinking them through, or you avoid making a decision at all because the stress has put you off your game. Find ways to manage your stress through mindfulness, exercise, yoga or doing something you enjoy.
- Give yourself some time (if possible). When you are under pressure, it’s hard to think clearly. Give yourself the chance to sit on a problem for a while so that you can process your options and feel confident about the course of action you choose.
- Weigh the pros and cons. When faced with a big decision, sometimes we lose sight of the big picture. Write a list of pros and cons for each course of action and then compare them.
- Think about your goals and values. It’s important to be true to ourselves and what we value in life. When you factor into a decision the things that are important to you, the best option might become obvious. At any rate, you’re more likely to end up with an outcome you’re happy with.
- Consider all the possibilities. Don’t just consider the positives and negatives of each option, as different consequences may result from your decision. When considering each option, also write down any likely consequences.
- Talk it out. It can be helpful to get another person’s perspective on your issue, particularly if they’ve faced a similar decision in their own life.
- Keep a diary. If you feel like you’re on a bit of an emotional rollercoaster, it might help to keep track of your feelings by writing them down.
- Plan how you’ll tell others. If you think someone may have a bad response to your decision, think through what their reaction is likely to be. Put yourself in their shoes to help you think of a good way to manage the situation.
- Rethink your options. If you’re up against a lot of pressure over a decision or there are some new factors to consider, look over your options again. You might decide that your original decision is still the best one, but give yourself the option of changing course. If a decision no longer feels right for you, go through these steps again to figure out a better solution.

If you’re having a tough time If you’re feeling overwhelmed with negative feelings because you’re facing a tough decision, it’s important to look after yourself. (See below for a link to self-care) If you’re finding that you’re needing extra help or your mental health is being affected, please access Student safety and wellbeing services (9905 3020 or monash.edu/counselling)

10 min: Give students each of these ‘What if …?’ hypothetical scenarios and ask them to come up with a “Plan B” (adapted from https://schools.au.reachout.com/resilience/flexible-thinking) (accessed on 7 September 2025):

- What if you were working on an assignment at home and realised you didn’t save the correct version of the assignment?
- What if you were working at a community pharmacy and realised you gave medications to the wrong patient?
- What if you were working at a hospital pharmacy and realised you can’t access the pathology results required for checking doses?

For your next reflection: Reflect upon a time where you had to think flexibly or adapt in your community pharmacy or hospital pharmacy placement.

- What went wrong? What didn’t go according to plan?
- What decision did you have to make?
- What was your “plan B”? If you didn’t have an alternative plan, what would you do differently now?

Self-care link: https://au.reachout.com/mental-wellbeing/self-care (accessed on 7 September 2025):

## Appendix E

P4

Building resilience through mistakes

Community pharmacy dispensing error case study

For Facilitators to give context to the case study:

Box A7 Facilitator Note: Decision-Making and Resilience Scenario. This is a community pharmacy intern who kept an audio diary and shares her experience after she made a dispensing error. She was in charge of packing for a Webster pack patient, and the intern needed to pack in a hurry, and she made a mistake by packing a duplicate of his blood pressure medications. Instead of packing Amlodipine with Olmetec Plus (olmesartan + hydrochlorothiazide), she packed Amlodipine with Sevikar Plus (amlodipine + olmesartan + hydrochlorothiazide).

5–10 min: Have students take turns and read together out loud this transcript and discuss the questions below:

“The reason being that I think this mistake happened was a few things. One was the pressure I had felt, particularly because I was like, “Oh, he hasn’t had his tablets for a couple of days, we really need to make sure these tablets are ready for him when he does come in because he said he was coming in.” And he didn’t really give a timeframe. He was a bit vague. So I thought, oh, what if he’s here in five minutes? I really want to make sure it’s ready. So I think that time pressure was one thing that happened.

And then another thing was the Sevikar that was in there; it was a generic brand. So it said olmesartan and amlodipine HCT. But I only read the olmesartan. And then I thought, “Ah yeah, that’s the one, pop it in.”

I didn’t double-check. So in hindsight. And I know we learn about that a lot, as well as that you’ve got to double-check your work. And I always hear that, but I guess I’m still a little bit in that frame of mind of “ah, the pharmacists are going to check my work. So I don’t really need to double check, somebody else will double check for me.” So I think that might be the thought process. I was having a little bit there too, that I haven’t quite switched over into that double-check my work frame of mind.

And I guess the biggest thing that I found upon reflection about this one was that they don’t teach you at uni how to cope with the emotional aspect of realizing you’ve made a mistake, because they can’t really teach you that it’s not something that can be taught how to cope with the emotions. But as soon as I realised.... All the swear words were going through my mind; basically, I was just so annoyed at myself for not realising that, and then the emotion of panic came through. And I was trying to call him to make sure that we could notify the patient as soon as possible. And he wouldn’t answer the phone.

We eventually got through to the patient, and I was quite emotional at that point. I had cried and, well, firstly, had held back tears earlier, and then about an hour later, I couldn’t hold back these tears anymore. Because I was very emotional about what had happened. Because just the worst thing possible to me is thinking of what could have happened ...that the unknown was really what was the most scary to me is this man, I don’t know what’s happened to him. “Has he taken the tablets?” “Has he had any severe consequences as a result?”

But anyway, then eventually, he called back, and then I got my manager to take the phone call for me. I thought I’d had enough new experiences today. I thought today, I might get an experienced pharmacist to handle the situation a little bit further. And then on the phone, the patient said, and I quote, “Oh, well, it won’t kill me.” So ...he was quite laid back about it, which, again, was quite lucky, not that we want this situation to happen. But it is lucky that this was my first experience. It wasn’t; he didn’t feel any issues with it. He was not all that fussed about it, and that kind of thing. But then I logged some incident reports, and I logged with PDL, as well.

So I learnt a lot of new things and things that we’ve definitely spoken about at uni about how to manage errors, etc. But I think going through the process itself is so different, because when in real life, it was quite a roller coaster of emotions. And my manager and preceptor afterwards, while at the time and afterwards, I’ve spoken to both of them. But they were saying that they were in a way glad I was upset by it, because they said that shows that I care about what I’m doing and that this will actually stick with me as an important thing to double check your work and not just rely on the pharmacist and things like that just to ensure that you won’t just brush it off and not care and then make the same mistake again, it will be a learning moment, a teaching moment. And yes, it will, in the end, be for the better. And that’s the thing we learn from our mistakes. It’s not, and if you don’t make mistakes, you don’t learn. I think in some ways, you just hope that the mistakes do get caught out by the pharmacist, or if you make a mistake, you find it yourself, for example, when you are practising as a pharmacist.

In time, I will look at it as something that I’ve learned from and hopefully something I will never repeat that mistake again. And I know maybe next time I’ll be a bit better at controlling my emotions, or not necessarily not feeling bad about it, because you still, of course, want to feel an emotion towards it so that you do understand the gravity of it. But more so to have a level-headed, critical thinking of problem solving of what to do next and how am I going to fix this, rather than “Oh God, I’ve done something wrong.”

1. How did this intern demonstrate resilience? (5–10 min)

Box A8 Facilitator Note: Learning from Mistakes and Seeking Support. For the facilitators, examples of what could be discussed:

- Viewed this experience as a way to learn and improve,/learnt from mistakes
- Ability to reflect upon mistakes and bounce back
- Being more aware of the future, remaining realistic about what could potentially happen in the future
- Asked for help and support from pharmacists/mentors

2. How could this intern have handled the situation better? (5–10 min)

Box A9 Facilitator Note: Responding Calmly After an Error. For the facilitators, examples of what could be discussed:

- Control emotions, managing stress/anxiety levels (mindfulness, simple breathing exercises)
- Be more level-headed, thinking critically about the situation
- Problem-solve on the issue at hand more calmly
- Accepting the error already made and concentrating on the next steps to minimise harm to the patient
- Instead of talking negatively to oneself (blaming or dwelling on how this mistake could have been made), think optimistically

Characteristics of resilient health professionals, according to *Matheson* et al., included optimism, flexibility and adaptability, initiative, tolerance, organisational skills, being a team worker, keeping within professional boundaries, confidence/assertiveness, humour, and a sense of self-worth.

3. What can be done to better prepare for these situations? Prevent them? Or are they inevitable? (5–10 min)

Box A10 Facilitator Note: Preventing Future Errors and Taking Responsibility. For the facilitators, examples of what could be discussed:

- Double-check your work
- Taking responsibility and not relying on others to pick up errors on your behalf
- Sharing with others what occurred so others don’t make the same mistake
- Not letting time pressure affect your work, setting clear timelines/prioritising work
- Having self-awareness that mistakes will happen, and knowing how to manage/problem solve, is more important than dwelling on the mistake itself

Next reflection for building resilience:

Reflect upon a time when you’ve made a mistake either at your workplace or at University.

How did you deal with the mistake? What could you have done differently? How could you have demonstrated resilience as you reflect upon the situation?

Resource:

Matheson C, Robertson HD, Elliott AM, Iversen L and Murchie P, 2016, Resilience of primary healthcare professionals working in challenging environments: a focus group study, British Journal of General Practice, 66 (648), Accessed via: https://bjgp.org/content/66/648/e507 (accessed on 9 June 2025).
